# Neonatal retroauricular cellulitis as an indicator of group B streptococcal bacteremia: a case report

**DOI:** 10.1186/1752-1947-3-9334

**Published:** 2009-12-16

**Authors:** David Pérez Solís, Juan José Díaz Martín, Etelvina Suárez Menéndez

**Affiliations:** 1 Department of Pediatrics, Hospital San Agustín, Camino de Heros, 4 33400 Avilés, Spain

## Abstract

**Introduction:**

The relation between cellulitis and Group B streptococcus infection in newborns and small infants was first reported during the early 1980s and named cellulitis-adenitis syndrome. We report a case of a neonate with cellulitis-adenitis syndrome in an unusual location (retroauricular).

**Case presentation:**

A 21-day-old Caucasian female infant was brought to the emergency department with fever, irritability and a decreased appetite. Physical examination revealed erythema and painful, mild swelling in the right retroauricular region. The blood count and C-reactive protein level were normal. She was treated with ceftriaxone. The fever and irritability were resolved after 24 hours, and the cellulitis was clearly reduced after two days of hospitalization. Blood culture yielded Group B streptococcus.

**Conclusion:**

A thorough evaluation must be done, and lumbar punctures for infants with cellulitis must be considered. We emphasize the lack of data about acute phase reactants to predict bacteremia and meningitis and to adjust the duration of parenteral antibiotic therapy to address this syndrome.

## Introduction

Group B streptococcus (GBS, Streptococcus agalactiae) is usually related to early onset neonatal sepsis, but it is also a cause of infection in neonates aged more than one week. The late onset of GBS infections normally manifest as sepsis, meningitis or, less frequently, focal infection [1].

The relation between cellulitis--with or without regional lymphadenitis--and GBS infections in newborns and small infants was first reported during the early 1980s. It was then named cellulitis-adenitis syndrome [2-4]. Cases described in the literature since then not only suggest that GBS bacteremia is common, but that meningeal involvement is also frequent [5]. Cellulitis is mostly located in the submandibular and preauricular area of the head [2]. We report the case of a neonate with retroauricular cellulitis without lymphadenitis. The results reveal that the neonate had GBS bacteremia.

## Case presentation

A 21-day-old Caucasian female infant from Spain was brought to our emergency department with a fever, irritability, and decreased appetite for six hours. She was born through vaginal delivery after 39 weeks of uncomplicated gestation. A vaginal culture of GBS was negative. The infant was bottle-fed since birth.

**Figure 1:**
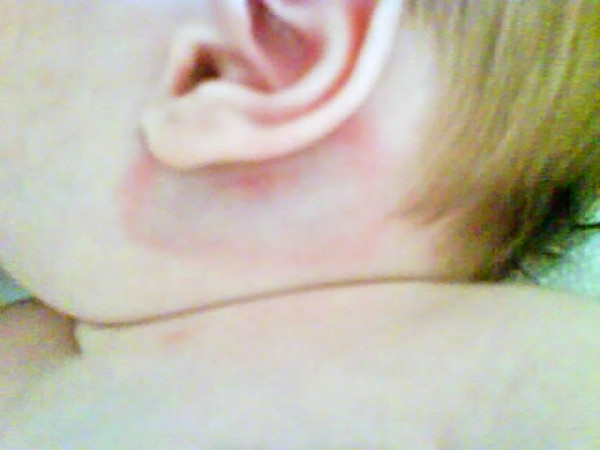
**Erythema and swelling in the right retroauricular region**.

The physical examination revealed that the infant had a fever (rectal temperature of 39.1°C) with erythema and painful but mild swelling in the right retroauricular region (Figure 1). The blood count revealed 5800 leukocytes/mm^3 ^(46% polymorphonuclear neutrophils and 1% bands), and hemoglobin 13.3 g/dL; the C-reactive protein level (CRP) was 5 mg/L; and the serum glucose and electrolytes, as well as urinalysis, were normal. A lumbar puncture was not performed.

She was admitted and treated empirically with ceftriaxone (75 mg/kg/day). The fever and irritability were resolved after 24 hours, and the cellulitis clearly improved after two days of hospitalization. Three days after admission, the blood culture yielded GBS. The infant was discharged after five days of treatment with ceftriaxone. Then, antibiotic therapy was continued with oral cephuroxime for the next seven days. An outpatient visit two weeks after discharge revealed no sequelae.

## Discussion

Late onset GBS infections usually occur between the ages of one week and three months. But, in up to one out of five cases, GBS infections may occur in infants older than three months of age [1].

In 1982, Baker [2] discussed GBS cellulitis-adenitis syndrome based on her own experience as well as that of other previously reported patients (a total of 16 cases). Her study showed that infants from two to 10 weeks of age suffer from a typical but abrupt onset of a fever, as well as poor feeding and/or irritability. Cellulitis was predominantly located in the submandibular region. But, in isolated cases, cellulitis was found in preauricular, cervical, genian, or inguinal regions. Adenitis was present in each infant with submandibular cellulitis. In 15 out of 16 patients, GBS bacteremia was present upon admission. Since then, some new cases with very similar features have been reported in the literature [6-12].

It has been suggested that subcutaneous infection is secondary to GBS bacteremia in infants with a previous skin or mucous colonization. Probably, certain subcutaneous areas are predisposed to becoming metastatic sites of infection. Another hypothesis is that bacteremia is secondary to a primary focus and lymphatic spread [2,3].

GBS cellulitis-adenitis syndrome is relevant because it is often associated with bacteremia and meningitis (91% and 24% of cases, respectively, according to a recent review [5]). Meningitis has been found even in infants in good clinical condition and with no clinical signs of central nervous system infection. Routine use of lumbar punctures is usually recommended in small infants with cellulitis-adenitis syndrome [5]. However, it must be noted that these are only isolated clinical cases. These incidences must not be overvalued, since published clinical cases are usually the most severe ones. It is more probable that lumbar punctures are performed on infants with worse clinical conditions. On the other hand, there is no data about the value of diagnostic tests (white blood cell count, C-reactive protein, procalcitonin, etc.) to predict bacteremia or meningitis in newborns and small infants with cellulitis. As a lumbar puncture was not performed on our patient, it is not possible for us to definitely rule out meningitis.

Antimicrobial therapy in patients with cellulitis-adenitis syndrome traditionally includes parenteral antibiotics for 10 to 14 days. Nowadays, the duration of the antimicrobial therapy may be guided by clinical and patient responses to acute phase reactants (especially C-reactive protein) [13].

## Conclusion

In our case, we emphasize the absence of adenitis in the retroauricular location even though our patient had GBS bacteremia, as with most cellulitis-adenitis cases. We conclude that for any newborn or small infant with cellulitis, a thorough evaluation must be done regardless of clinical condition. A lumbar puncture must also be considered. It would be interesting to have available studies on the global incidence of GBS bacteremia and meningitis in cellulitis-adenitis syndrome, as well as on the value of acute phase reactants to predict them and to adjust the duration of parenteral antibiotic therapy.

## Abbreviations

CRP: C-reactive protein; GBS: Group B streptococcus.

## Consent

Written informed parental consent was obtained for both print and online publication of this case and any accompanying images. A copy of the written consent is available for review by the Editor-in-Chief of this journal.

## Competing interests

The authors declare that they have no competing interests.

## Authors' contributions

DPS was a major contributor in writing the manuscript and preparing the literature review. JJDM and ESM interpreted the patient data and were contributors in writing the manuscript. All authors read and approved the final manuscript.
